# Predicting gene function using hierarchical multi-label decision tree ensembles

**DOI:** 10.1186/1471-2105-11-2

**Published:** 2010-01-02

**Authors:** Leander Schietgat, Celine Vens, Jan Struyf, Hendrik Blockeel, Dragi Kocev, Sašo Džeroski

**Affiliations:** 1 Department of Computer Science, Katholieke Universiteit Leuven, Celestijnenlaan 200A, 3001 Leuven, Belgium; 2 Department of Knowledge Technologies, Jožef Stefan Institute, Jamova cesta 39, 1000 Ljubljana, Slovenia

## Abstract

**Background:**

*S. cerevisiae*, *A. thaliana *and *M. musculus *are well-studied organisms in biology and the sequencing of their genomes was completed many years ago. It is still a challenge, however, to develop methods that assign biological functions to the ORFs in these genomes automatically. Different machine learning methods have been proposed to this end, but it remains unclear which method is to be preferred in terms of predictive performance, efficiency and usability.

**Results:**

We study the use of decision tree based models for predicting the multiple functions of ORFs. First, we describe an algorithm for learning hierarchical multi-label decision trees. These can simultaneously predict all the functions of an ORF, while respecting a given hierarchy of gene functions (such as FunCat or GO). We present new results obtained with this algorithm, showing that the trees found by it exhibit clearly better predictive performance than the trees found by previously described methods. Nevertheless, the predictive performance of individual trees is lower than that of some recently proposed statistical learning methods. We show that ensembles of such trees are more accurate than single trees and are competitive with state-of-the-art statistical learning and functional linkage methods. Moreover, the ensemble method is computationally efficient and easy to use.

**Conclusions:**

Our results suggest that decision tree based methods are a state-of-the-art, efficient and easy-to-use approach to ORF function prediction.

## Background

The completion of several genome projects in the past decade has generated the full genome sequence of many organisms. Identifying open reading frames (ORFs) in the sequences and assigning biological functions to them has now become a key challenge in modern biology. This last step, which is the focus of our paper, is often guided by automatic discovery processes which interact with the laboratory experiments.

More precisely, machine learning techniques are used to predict gene functions from a predefined set of possible functions (e.g., the functions in the Gene Ontology). Afterwards, the predictions with highest confidence can be tested in the lab. There are two characteristics of the function prediction task that distinguish it from common machine learning tasks: (1) a single gene may have multiple functions; and (2) the functions are organized in a hierarchy: a gene that is related to some function is automatically related to all its ancestor functions (this is called the hierarchy constraint). This particular problem setting is known in machine learning as hierarchical multi-label classification (HMC) and recently, many approaches have been proposed to deal with it [1-19]. These approaches differ with respect to a number of characteristics: which learning algorithm they are based on, whether the hierarchy constraint is always met and whether they can deal with hierarchies structured as a directed acyclic graph (DAG), such as the Gene Ontology, or are restricted to hierarchies structured as a rooted tree, like MIPS's FunCat.

Decision trees are a well-known type of classifiers that can be learned efficiently from large datasets, produce accurate predictions and can lead to knowledge that provides insight in the biology behind the predictions, as demonstrated by Clare et al. [3]. They have been applied to several machine learning tasks [20]. In earlier work [14], we have investigated how they can be extended to the HMC setting: we presented an HMC decision tree learner that takes into account the hierarchy constraint and that is able to process DAG structured hierarchies.

In this article, we show that our HMC decision tree method outperforms previously published approaches applied to *S. cerevisiae *and *A. thaliana*. Our comparisons primarily use precision-recall curves. This evaluation method is well-suited for the HMC tasks considered here, due to the large class skew present in these tasks.

Moreover, we show that by upgrading our method to an ensemble technique, classification performance improves further. Ensemble techniques are learning methods that construct a set of classifiers and classify new data instances by taking a vote over their predictions. Experiments show that ensembles of decision trees outperform Bayesian corrected support vector machines [10], a statistical learning method for gene function prediction, on *S. cerevisiae *data, and methods participating in the MouseFunc challenge [21,22] on *M. musculus *data.

### Related work

A number of machine learning approaches have been proposed in the area of functional genomics. They have been applied in the context of gene function prediction in *S. cerevisiae*, *A. thaliana *or *M. musculus*. We have grouped them according to the learning approach they use.

#### Network based methods

Several approaches predict functions of unannotated genes based on known functions of genes that are nearby in a functional association network or protein-protein interaction network [2,4,5,8,15-17]. GENEFAS [4], for example, predicts functions of unannotated yeast genes based on known functions of genes that are nearby in a functional association network. GENEMANIA [15] calculates per gene function a composite functional association network from multiple networks derived from different genomic and proteomic data sources.

These approaches are based on label propagation and do not return a global predictive model. However, a number of approaches were proposed to combine predictions of functional networks with those of a predictive model. Kim et al. [16] combine them with predictions from a Naive Bayes classifier. The combination is based on a simple aggregation function. The Funckenstein system [17] uses logistic regression to combine predictions made by a functional association network with predictions from a random forest.

#### Kernel based methods

Deng et al. [1] predict gene functions with Markov random fields using protein interaction data. They learn a model for each gene function separately and ignore the hierarchical relationships between the functions. Lanckriet et al. [6] represent the data by means of a kernel function and construct support vector machines for each gene function separately. They only predict top-level classes in the hierarchy. Lee et al. [13] have combined the Markov random field approach of [1] with the SVM approach of [6] by computing diffusion kernels and using them in kernel logistic regression.

Obozinski et al. [19] present a two-step approach in which SVMs are first learned independently for each gene function separately (allowing violations of the hierarchy constraint) and are then reconciliated to enforce the hierarchy constraint. Barutcuoglu et al. [10] have proposed a similar approach where unthresholded support vector machines are learned for each gene function and then combined using a Bayesian network so that the predictions are consistent with the hierarchical relationships. Guan et al. [18] extend this method to an ensemble framework that is based on three classifiers: a classifier that learns a single support vector machine for each gene function, the Bayesian corrected combination of support vector machines mentioned above, and a classifier that constructs a single support vector machine per gene function and per data source and forms a Naive Bayes combination over the data sources.

Methods that learn a separate model for each function have several disadvantages. Firstly, they are less efficient, because *n *models have to be built (with *n *the number of functions). Secondly, they often learn from strongly skewed class distributions, which is difficult for many learners.

#### Decision tree based methods

Clare [23] presents an HMC decision tree method that learns a single tree for predicting gene functions of *S. cerevisiae*. She adapts the well-known decision tree algorithm C4.5 [20] to cope with the issues introduced by the HMC task. First, where C4.5 normally uses class entropy for choosing the best split, her version uses the sum of entropies of the class variables. Second, she extends the method to predict classes on several levels of the hierarchy, assigning a larger cost to misclassifications higher up in the hierarchy. The resulting tree is transformed into a set of rules, and the best rules are selected, based on a significance test performed on a separate validation set. Note that this last step violates the hierarchy constraint, since rules predicting a class can be dropped while rules predicting its subclasses are kept. The non-hierarchical version of her method was later used to predict GO terms for *A. thaliana *[9]. Here, the annotations are predicted for each level of the hierarchy separately.

Hayete and Bienkowska [7] build a decision tree for each GO function separately using information about protein assignments in the same functional domain. As mentioned earlier, methods that learn separate models for each function have several disadvantages. Moreover, Vens et al. [14] show that in the context of decision trees, separate models are less accurate than a single HMC tree that predicts all functions at once.

Blockeel et al. [24] present to our knowledge the first decision tree approach to HMC that exploits the given class hierarchy and predicts all classes with a single decision tree. Their method is based on the predictive clustering tree framework [25]. This method was first applied to gene function prediction by Struyf et al. [26]. Later, Blockeel et al. [27] propose an improved version of the method and evaluate it on yeast functional genomics data. Vens et al. [14] extend the algorithm towards hierarchies structured as DAGs and show that learning one decision tree for simultaneously predicting all functions outperforms learning one tree per function (even if those trees are built taking into account the hierarchy).

## Methods

We first discuss the approach to building HMC trees presented in [14] and then extend it to build ensembles of such trees.

### Using predictive clustering trees for HMC tasks

The approach that we present is based on decision trees and is set in the predictive clustering tree (PCT) framework [25]. This framework views a decision tree as a hierarchy of clusters: the top-node corresponds to one cluster containing all training examples, which is recursively partitioned into smaller clusters while moving down the tree. PCTs can be applied to both clustering and prediction tasks. The PCT framework is implemented in the CLUS system, which is available at http://www.cs.kuleuven.be/~dtai/clus.

**Figure 1:**
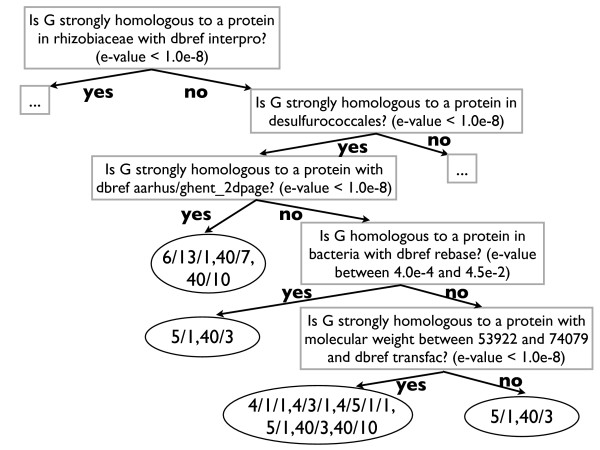
**Example of a predictive clustering tree**. This tree predicts the functions of a gene *G*, based on homology data. The functions are taken from the FunCat classification scheme and are hierarchical: if for example function 4/3/1 (tRNA synthesis) is predicted, then function 4/3 (tRNA transcription) and function 4 (transcription) are predicted as well.

Before explaining the approach in detail, we show an example of a (partial) predictive clustering tree predicting the functions of *S. cerevisiae *genes from homology data [23] (Figure 1). The homology features are based on a sequence similarity search performed for each yeast gene against all the genes in SwissProt. The functions are taken from the FunCat classification scheme [28]. Each internal node of the tree contains a test on one of the attributes in the dataset. Here, the attributes are binary and have been obtained after preprocessing the relational homology data with a frequent pattern miner. The root node, for instance, tests whether there exists a SwissProt protein that has a high similarity (e-value < 1.0·10^-8^) with the gene under consideration *G*, is classified into the rhizobiaceae group and has references to the InterPro database. In order to predict the functions of a new gene, the gene is routed down the tree according to the outcome of the tests. When a leaf node is reached, the gene is assigned the functions that are stored in it. Only the most specific functions are shown in the figure. In the rest of this section, we explain how PCTs are constructed. A detailed explanation is given in [14].

PCTs [25] can be constructed with a standard "top-down induction of decision trees" (TDIDT) algorithm, similar to CART[29] or C4.5 [20]. The algorithm takes as input a set of training instances (i.e., the genes and their annotations). It searches for the best acceptable test that can be put in a node. If such a test can be found then the algorithm creates a new internal node and calls itself recursively to construct a subtree for each subset (cluster) in the partition induced by the test on the training instances. To select the best test, the algorithm scores the tests by the reduction in variance (which is defined below) they induce on the instances. Maximizing variance reduction maximizes cluster homogeneity and improves predictive performance. If no acceptable test can be found, that is, if no test significantly reduces variance (as measured by a statistical *F*-test), then the algorithm creates a leaf and labels it with a representative case, or prototype, of the given instances.

To apply PCTs to the task of hierarchical multi-label classification, the variance and prototype are defined as follows [14].

**Figure 2:**
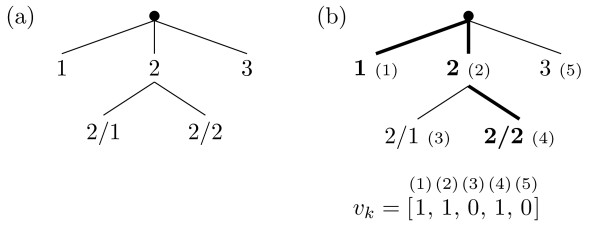
**A toy hierarchy**. (a) Class label names reflect the position in the hierarchy, e.g., '2/1' is a subclass of '2'. (b) The set of classes {1,2,2/2}, indicated in bold in the hierarchy, and represented as the vector *v*_*k*_.

First, the set of labels of each example is represented as a vector with binary components; the *i*'th component of the vector is 1 if the example belongs to class *c*_*i *_and 0 otherwise. It is easily checked that the arithmetic mean of a set of such vectors contains as *i*'th component the proportion of examples of the set belonging to class *c*_*i*_. We define the variance of a set of examples *S *as the average squared distance between each example's class vector *v*_*k *_and the set's mean class vector , i.e.,

In the HMC context, it makes sense to consider similarity at higher levels of the hierarchy more important than similarity at lower levels. To that aim, we use a weighted Euclidean distance

where *v*_*k*, *i *_is the *i*'th component of the class vector *v*_*k *_of an instance *x*_*k*_, and the class weights *w*(*c*) decrease with the depth of the class in the hierarchy. We choose *w*(*c*) = *w*_0_·avg_j _{*w*(*p*_*j*_(*c*))}, where *p*_*j *_(*c*) denotes the *j*'th parent of class *c *and 0 <*w*_0 _< 1). Consider, for example, the class hierarchy shown in Figure 2, and two examples (*x*_1_, *S*_1_) and (*x*_2_, *S*_2_) with *S*_1 _= {1, 2, 2/2} and *S*_2 _= {2}. Using a vector representation with consecutive components representing membership of class 1, 2, 2/1, 2/2 and 3, in that order, we have

The heuristic for choosing the best test for a node of the tree is to maximize the variance reduction as discussed before, with the above definition of variance. Note that our definition of *w*(*c*) allows the classes to be structured in a DAG, as is the case with the Gene Ontology.

Second, a classification tree stores in a leaf the majority class for that leaf; this class will be the tree's prediction for examples arriving in the leaf. But in our case, since an example may have multiple classes, the notion of "majority class" does not apply in a straightforward manner. Instead, the mean  of the class vectors of the examples in that leaf is stored. Recall that  is the proportion of examples in the leaf belonging to *c*_*i*_. An example arriving in the leaf can therefore be predicted to belong to class *c*_*i *_if  is above some threshold *t*_*i*_, which can be chosen by the user. To ensure that the predictions obey the hierarchy constraint (whenever a class is predicted its superclasses are also predicted), it suffices to choose *t*_*i *_≤ *t*_*j *_whenever *c*_*i *_is a superclass of *c*_*j *_. The PCT in Figure 1 has a threshold of *t*_*i *_= 0.4 for all *i*.

CLUS-HMC is the instantiation (with the distances and prototypes defined as above) of the PCT algorithm implemented in the CLUS system.

### Ensembles of PCTs

Ensemble methods are learning methods that construct a set of classifiers for a given prediction task and classify new examples by combining the predictions of each classifier. In this paper we consider bagging, an ensemble learning technique that has primarily been used in the context of decision trees. In preliminary experiments, we also considered two other ensemble learning techniques: random forests [30] and an adapted version of the boosting approach for regression trees by Drucker [31]. However, neither method performed better than simple bagging.

Bagging [32] is an ensemble method where the different classifiers are constructed by making bootstrap replicates of the training set and using each of these replicates to construct one classifier. Each bootstrap sample is obtained by randomly sampling training instances, with replacement, from the original training set, until the sample contains the same number of instances as the original training set. The individual predictions given by each classifier can be combined by taking the average (for numeric targets) or the majority vote (for nominal targets).

Breiman has shown that bagging can give substantial gains in the predictive performance of decision tree learners [32]. Also in the case of learning PCTs for predicting multiple targets at once (multi-task learning [33]), decision tree methods benefit from the application of bagging [34]. However, it is clear that, by using bagging on top of the PCT algorithm, the learning time of the model increases significantly, resulting in a clear trade-off between predictive performance and efficiency to be considered by the user.

The algorithm for bagging PCTs takes as input the parameter *k*, denoting the number of trees in the ensemble. In order to make predictions, the average of all class vectors predicted by the *k *trees in the ensemble is computed, and then the threshold is applied as before. This ensures that the hierarchy constraint holds. We call the resulting instantiation of the bagging algorithm around the CLUS-HMC algorithm CLUS-HMC-ENS.

## Results and discussion

In this section, we address the following questions:

1. How well does CLUS-HMC perform on functional genomics data and what is the improvement, if any, that can be obtained by using CLUS-HMC-ENS on such tasks?

2. How does the predictive performance of the proposed algorithms compare to results reported in the biomedical literature?

In order to answer these questions, we compare our results to the results reported by Clare and King [3] and Barutcuoglu et al. [10] on *S. cerevisiae*, to the results reported by Clare et al. [9] on *A. thaliana*, and to the results of the groups participating in the MouseFunc challenge [21,22] on *M. musculus*. The methods used in these studies were discussed in the "Related work" section.

### Datasets

For *S. cerevisiae *and *A. thaliana*, the datasets that we use in our evaluation are exactly those datasets that are used in the cited articles. They are available, together with the parameter settings that can be used to reproduce the results, at the following webpage: http://www.cs.kuleuven.be/~dtai/clus/hmc-ens. For *M. musculus*, the (raw) data is available at http://hugheslab.med.utoronto.ca/supplementary-data/mouseFunc_I/, while the dataset we assembled from it is available at the former webpage.

Next to predicting gene functions of three organisms (*S. cerevisiae*, *A. thaliana*, and *M. musculus*), we consider two annotation schemes in our evaluation: FunCat (developed by MIPS [28]), which is a tree-structured class hierarchy and the Gene Ontology (GO) [35], which forms a directed acyclic graph instead of a tree: each term can have multiple parents.

#### Saccharomyces cerevisiae

The first dataset we use (**D**_**0**_) was described by Barutcuoglu et al. [10] and is a combination of different data sources. The input feature vector for a gene consists of pairwise interaction information, membership to colocalization locale, possession of transcription factor binding sites and results from microarray experiments, yielding a dataset with in total 5930 features. The 3465 genes are annotated with function terms from a subset of 105 nodes from the Gene Ontology's *biological process *hierarchy.

We also use the 12 yeast datasets (**D**_**1 **_- **D**_**12**_) from [23]. The datasets describe different aspects of the genes in the yeast genome. They include five types of bioinformatics data: sequence statistics, phenotype, secondary structure, homology and expression. The different sources of data highlight different aspects of gene function. The genes are annotated with functions from the FunCat classification schemes. Only annotations from the first four levels are given.

**D**_**1 **_(seq) records sequence statistics that depend on the amino acid sequence of the protein for which the gene codes. These include amino acid frequency ratios, sequence length, molecular weight and hydrophobicity.

**D**_**2 **_(pheno) contains phenotype data, which represents the growth or lack of growth of knock-out mutants that are missing the gene in question. The gene is removed or disabled and the resulting organism is grown with a variety of media to determine what the modified organism might be sensitive or resistant to.

**D**_**3 **_(struc) stores features computed from the secondary structure of the yeast proteins. The secondary structure is not known for all yeast genes; however, it can be predicted from the protein sequence with reasonable accuracy, using Prof [36]. Due to the relational nature of secondary structure data, Clare performed a preprocessing step of relational frequent pattern mining; *D*_3 _includes the constructed patterns as binary attributes.

**D**_**4 **_(hom) includes for each yeast gene, information from other, homologous genes. Homology is usually determined by sequence similarity; here, PSI-BLAST [37] was used to compare yeast genes both with other yeast genes and with all genes indexed in SwissProt v39. This provided for each yeast gene a list of homologous genes. For each of these, various properties were extracted (keywords, sequence length, names of databases they are listed in, ...). Clare preprocessed this data in a similar way as the secondary structure data to produce binary attributes.

**D**_**5**_, ..., **D**_**12**_. Many microarray datasets exist for yeast and several of these were used [23]. Attributes for these datasets are real valued, representing fold changes in expression levels.

#### Arabidopsis thaliana

We use six datasets from [9], originating from different sources: sequence statistics, expression, predicted SCOP class, predicted secondary structure, InterPro and homology. Each dataset comes in two versions: with annotations from the FunCat classification scheme and from the Gene Ontology's *molecular function *hierarchy. Again, only annotations for the first four levels are given. We use the manual annotations for both schemes.

**D**_**13 **_(seq) records sequence statistics in exactly the same way as for *S. cerevisiae*. **D**_**14 **_(exprindiv) contains 43 experiments from NASC's Affymetrix service "Affywatch" http://affymetrix.arabidopsis.info/AffyWatch.html, taking the signal, detection call and detection *p*-values. **D**_**15 **_(scop) consists of SCOP superfamily class predictions made by the Superfamily server [38]. **D**_**16 **_(struc) was obtained in the same way as for *S. cerevisiae*. **D**_**17 **_(interpro) includes features from several motif or signature finding databases, like PROSITE, PRINTS, Pfam, ProDom, SMART and TIGRFAMs, calculated using the EBI's stand-alone InterProScan package [39]. To obtain features, the relational data was mined in the same manner as the structure data. **D**_**18 **_(hom) was obtained in the same way as for *S. cerevisiae*, but now using SwissProt v41.

#### Mus musculus

We use the data that was provided for the MouseFunc challenge [21,22]. It consists of 21603 genes, of which 1718 are set aside as test genes. Each gene is annotated with GO terms from a specified subset of the Gene Ontology. The annotations are up-propagated using the Gene Ontology's "is-a" and "part-of" relation. The data is composed of several sources: gene expression data, protein sequence pattern annotations, protein-protein interactions, phenotype annotations, phylogenetic profile and disease associations. In order to construct a single dataset (**D**_**19**_), we joined all data tables, removed attributes with fewer than five non-zero values and computed additional attributes that indicate for each gene the classes of other genes to which it is linked through a protein-protein interaction (only considering training set genes). This yields 18746 attributes in total. The resulting representation is similar to the one used by Guan et al. [18].

### Methodology

#### Evaluation measure

We report the performance of the different methods with precision-recall (PR) and ROC [40] based evaluation measures. This is motivated by the following two observations: (1) both measures have been used before to evaluate approaches to gene function prediction [1,8,22], and (2) they both allow to simultaneously compare classifiers for different classification thresholds. Of both measures, PR based evaluation better suits the characteristics of typical HMC datasets, in which many classes are infrequent (i.e., typically only a few genes have a particular function). Viewed as a binary classification task for each class, this implies that for most classes the number of negative instances by far exceeds the number of positive instances. In some cases, it is preferred to recognize the positive instances (i.e., that a gene has a given function), rather than correctly predict the negative ones (i.e., that a gene does not have a particular function). ROC curves are then less suited for this task, exactly because they also reward a learner if it correctly predicts negative instances (giving rise to a low false positive rate). This can present an overly optimistic view of the algorithm's performance [41]. Therefore, unless it is impossible to reconstruct the PR behaviour of the methods we compare to, we report a PR based evaluation.

We use the following definitions of precision, recall, average precision, and average recall:

where *i *ranges over all functions, *T P*_*i *_is the number of true positives (correctly predicted positive instances) for function *i*, *F P*_*i *_is the number of false positives (positive predictions that are incorrect) for function *i*, and *F N*_*i *_is the number of false negatives (positive instances that are incorrectly predicted negative) for function *i*. Note that these measures ignore the number of correctly predicted negative examples.

A precision-recall curve (PR curve) plots the precision of a model as a function of its recall. We consider two types of PR curves: (1) a function-wise PR curve for a given function *i*, which plots *Precision*_*i *_versus *Recall*_*i*_, and (2) an average or pooled PR curve, which plots  versus  and summarizes the performance of the model across all functions.

We construct the PR curves as follows. Remember that every leaf in the tree contains a vector  with for each function the probability that the gene is predicted to have this function. When decreasing the prediction threshold *t*_*i *_from 1 to 0, an increasing number of instances is predicted to belong to *c*_*i*_, causing the recall to increase whereas precision may increase or decrease (with normally a tendency to decrease). Thus, a single tree (or an ensemble of trees) with a specific threshold has a single precision and recall, and by varying the threshold a PR curve is obtained. Such curves allow us to evaluate the predictive performance of a model regardless of *t*. In the end, a domain expert can choose the threshold corresponding to the point on the curve that looks most interesting to him.

Although a PR curve helps in understanding the predictive behaviour of the model, a single performance score is more useful to compare models. A score often used to this end is the area between the PR curve and the recall axis, the so-called "area under the PR curve" (AUPRC). The closer the AUPRC is to 1.0, the better the model is. We consider two measures that are based on this idea, that correspond to the two types of PR curves and that are often reported in the literature: AU(), the area under the average PR curve, and , the average over all areas under the function-wise PR curves. Note that AU() gives more weight to more frequent functions, while  considers the importance of every function to be equal.

#### Parameter settings for CLUS-HMC and CLUS-HMC-ENS

In the experiments, *w*_0_, which determines the weights of the different functions in the decision tree heuristic, is set to 0.75 and the number of examples in each decision tree leaf is lower bounded to 5. The parameter *k*, which denotes the number of trees used in the ensemble, is set to 50. Preliminary experiments show that performance does not strongly depend on the choice of *w*_0 _and that it does not significantly increase after *k *= 50, so the latter value is a good trade-off between performance and runtime. The significance parameter used in the *F*-test stopping criterion of CLUS-HMC and CLUS-HMC-ENS is tuned on a separate validation set (1/3 of the training data) and optimized out of 6 possible values (0.001, 0.005, 0.01, 0.05, 0.1, 0.125), maximizing the AU(). The final model is constructed on the entire training set using the selected value of the significance parameter.

### Results

We will first investigate if ensembles improve the predictive performance of CLUS-HMC in gene function prediction and if so, quantify this difference. We will then compare CLUS-HMC and CLUS-HMC-ENS against several state-of-the-art systems in gene function prediction. On the one hand, we will compare CLUS-HMC to C4.5H/M [3,9], because they both build a single decision tree. On the other hand, we will compare CLUS-HMC-ENS to Bayesian-corrected SVMs [10], a statistical learning approach, on *D*_0_, and to the methods that entered the MouseFunc challenge on *D*_19_.

The datasets originating from [3,9] (i.e., datasets *D*_1 _to *D*_18_) are divided into a training set (2/3) and a test set (1/3). We use exactly the same splits. For dataset *D*_0_, we randomly construct a training and test set with the same ratio. For dataset *D*_19_, we use the same training and test sets that were used in the MouseFunc challenge.

#### Comparison between CLUS-HMC and CLUS-HMC-ENS

**Figure 3:**
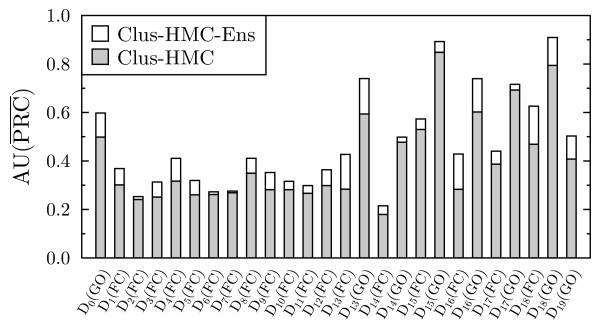
**Comparison of AU() between Clus-HMC and Clus-HMC-Ens**. The white surface represents the gain in AU() obtained by CLUS-HMC-ENS.

**Figure 4:**
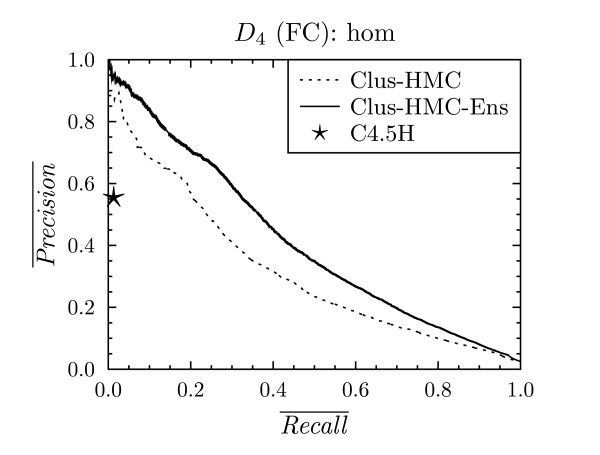
**Precision-recall curve for all classes for C4.5H, Clus-HMC and Clus-HMC-Ens on *D*_4 _with FunCat annotations**.

**Figure 5:**
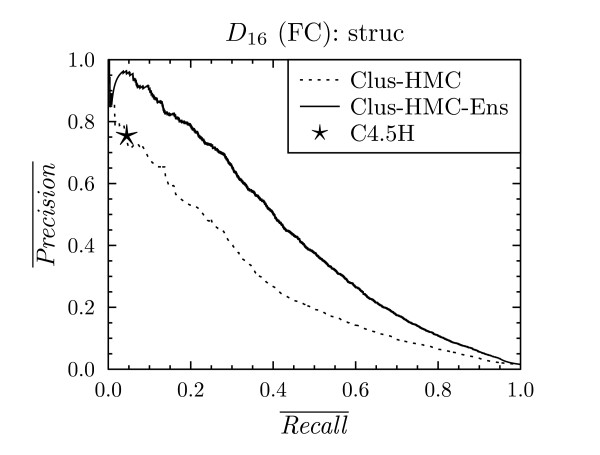
**Precision-recall curve for all classes for C4.5H, Clus-HMC and Clus-HMC-Ens on *D*_16 _with FunCat annotations**.

**Figure 6:**
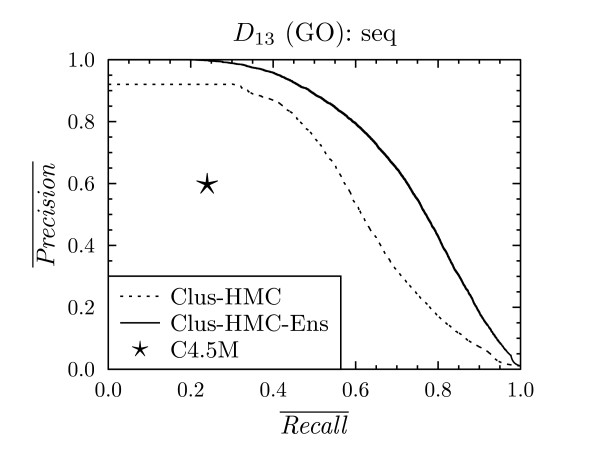
**Precision-recall curve for all classes for C4.5M, Clus-HMC and Clus-HMC-Ens on *D*_13 _with GO annotations**.

**Figure 7:**
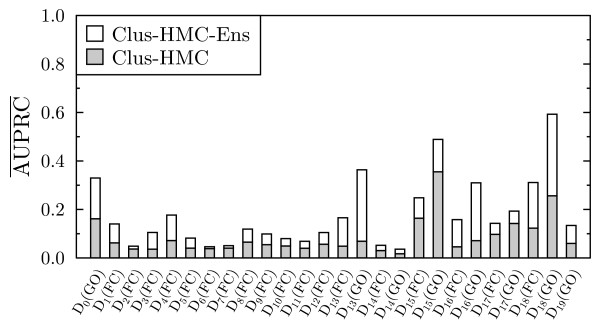
**Comparison of **** between Clus-HMC and Clus-HMC-Ens**. The white surface represents the gain in  obtained by CLUS-HMC-ENS.

For each of the datasets, the AU() of CLUS-HMC and CLUS-HMC-ENS is shown in Figure 3. We see that for every dataset, there is an increase in AU() when using ensembles. The average gain is 0.071 (which is an improvement of 18% on average); the maximal gain is 0.157. Representative PR curves can be found in Figures 4, 5 and 6. Figure 7 shows the  of CLUS-HMC and CLUS-HMC-ENS. Again, there is an increase in  when using ensembles, with an average gain of 0.093 (which is an improvement of 108% on average) and a maximal gain of 0.337. These results show that the increase in performance obtained by CLUS-HMC-ENS is larger according to  than according to AU(), which indicates that ensembles are performing particularly better for the less frequent classes, typically occurring at the lower levels of the hierarchy. To summarize, the improvement in predictive performance that can be obtained by using tree ensembles in more straightforward machine learning settings carries over to the HMC setting with functional genomics data.

#### Comparison between CLUS-HMC and C4.5H/M

We now concentrate on the comparison of the results obtained by our algorithms to those obtained by other decision tree based algorithms. For the datasets that are annotated with FunCat classes (*D*_1 _- *D*_18_), we will compare to the hierarchical extension of C4.5 [3], which we will refer to as C4.5H. For the datasets with GO annotations (*D*_13 _- *D*_18_), we will use the non-hierarchical multi-label extension of C4.5 [9], as C4.5H cannot handle hierarchies structured as a DAG. We refer to this system as C4.5M.

For their experiments on *A. thaliana*, Clare et al. [9] only report results per level of the hierarchy. In order to obtain these results, they learn a separate classifier per level, removing from their training and test set those genes that do not have annotated functions at that level. This approach may give a biased result: when annotating a new gene, it is not known in advance at which levels of the hierarchy it will have functions. Therefore, we reran C4.5M to learn one classifier that uses all training data and tested it on the complete test set.

For evaluating their systems, Clare et al. [3,9] report precision. Indeed, as the biological experiments required to validate the learned rules are costly, it is important to avoid false positives. However, precision is always traded off by recall: a classifier that predicts one example positive, but misses 1000 other positive examples may have a precision of 1, although it can hardly be called a good classifier. Therefore, we also compute the recall of the models obtained by C4.5H/M. These models were presented as rules for specific classes without any probability scores, so each model corresponds to precisely one point in PR space.

For each of the datasets *D*_1 _- *D*_18_, these PR points are plotted against the average PR curves for CLUS-HMC. As we are comparing curves with points, we speak of a "win" for CLUS-HMC when its curve is above C4.5H/M's point, and of a "loss" when it is below the point. Under the null hypothesis that both systems perform equally well, we expect as many wins as losses. We observed that only in one case out of 24, for dataset *D*_16 _with FunCat annotations, C4.5H/M outperforms CLUS-HMC. For all other cases there is a clear win for CLUS-HMC. Representative PR curves can be found in Figures 4, 5 and 6.

**Figure 8:**
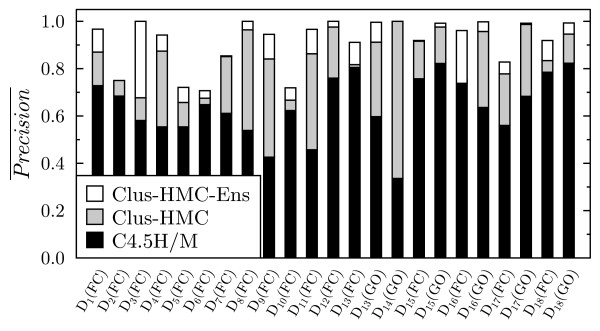
**Comparison of precision between C4.5H/M, Clus-HMC and Clus-HMC-Ens, at the recall obtained by C4.5H/M**. The gray surface represents the gain in precision obtained by CLUS-HMC, the white surface represents the gain for CLUS-HMC-ENS. *D*_14_(FC) was not included, since C4.5H did not find significant rules. For *D*_16_(FC), C4.5H scored a slightly better precision (see Figure 5), hence the lack of gray surface.

For each of these datasets, we also compared the precision of C4.5H/M, CLUS-HMC and CLUS-HMC-ENS, at the recall obtained by C4.5H/M. The results can be found in Figure 8. The average gain in precision w.r.t. C4.5H/M is 0.209 for CLUS-HMC and 0.276 for CLUS-HMC-ENS.

We can conclude that CLUS-HMC is the tree-building system that yields the best predictive performance. Compared with other existing methods, we are able to obtain the same precision with higher recall, or the same recall with higher precision. Moreover, the hierarchy constraint is always fulfilled, which is not the case for C4.5H/M.

#### Comparing individual rules

**Figure 9:**
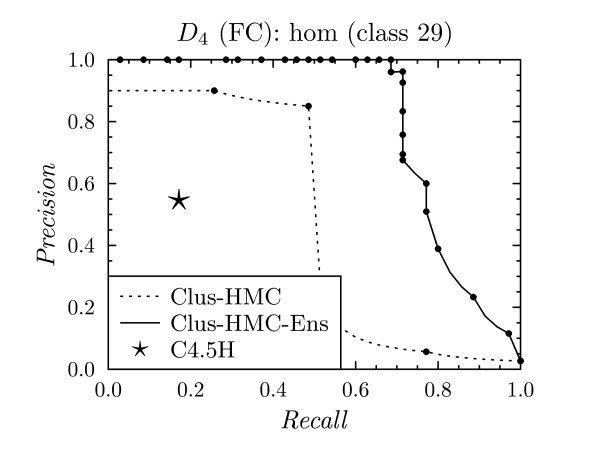
**Precision-recall curve for class 29 on *D*_4 _with FunCat annotations**.

**Figure 10:**
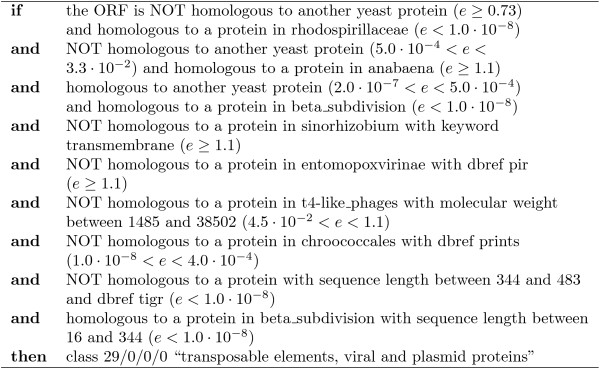
**Rule found by C4.5H on the *D*_4 _(FC) homology dataset, with a precision of 0.55 and a recall of 0.17**.

**Figure 11:**
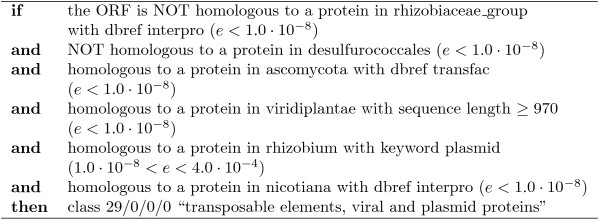
**Rule found by Clus-HMC on the *D*_4 _(FC) homology dataset, with a precision of 0.90 and a recall of 0.26**.

Every leaf of a decision tree corresponds to an *if *... *then *... rule. When comparing the complexity and precision/recall of these individual rules, CLUS-HMC also performs well. For instance, take FunCat class 29, which has a prior frequency of 3%. Figure 9 shows the PR evaluation for the algorithms for this class using homology dataset *D*_4_. The PR point for C4.5H corresponds to one rule, shown in Figure 10. This rule has a precision/recall of 0.55/0.17. CLUS-HMC's most precise rule for class 29 is shown in Figure 11. This rule has a precision/recall of 0.90/0.26.

Note from Figure 9 that an even higher precision can be obtained with CLUS-HMC-ENS, although the rules which lead to this prediction are more complex.

#### Comparison between CLUS-HMC-ENS and Bayesian-corrected SVMs

In this section, we compare CLUS-HMC-ENS to the statistical learning method of Barutcuoglu et al. [10], which consists of Bayesian-corrected SVMs (see "Related work"). We will further refer to this method as BSVM. The authors have used dataset *D*_0 _to evaluate their method and report class-wise area under the ROC convex hull (AUROC) for a small subset of 105 nodes of the Gene Ontology. As only AUROC scores are reported by Barutcuoglu et al. [10], we adopt the same evaluation metric for this comparison.

Barutcuoglu et al. [10] build a bagging procedure around their system and report out-of-bag error estimates [42] as evaluation, which removes the need for a set-aside test set. Out-of-bag error estimation proceeds as follows: for each example in the original training set, the predictions are made by aggregating only over those classifiers for which the example was not used for training. This is the out-of-bag classifier. The out-of-bag error estimate is then the error rate of the out-of-bag classifier on the training set. The number of bags used in this procedure was 10. To compare our results, we use exactly the same method.

**Figure 12:**
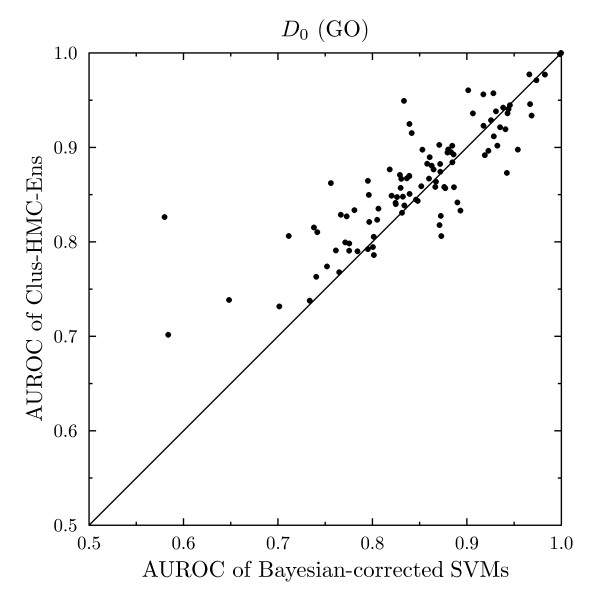
**Class-wise out-of-bag AUROC comparison between Clus-HMC-Ens and Bayesian-corrected SVMs**.

On dataset *D*_0_, the average of the AUROC over the 105 functions is 0.871 for CLUS-HMC-ENS and 0.854 for BSVM. Figure 12 compares the class-wise out-of-bag AUROC estimates for CLUS-HMC-ENS and BSVM outputs. CLUS-HMC-ENS scores better on 73 of the 105 functions, while BSVM scores better on the remaining 32 cases. According to the (two-sided) Wilcoxon signed rank test [43], the performance of CLUS-HMC-ENS is significantly better (*p *= 4.37·10^-5^).

Moreover, CLUS-HMC-ENS is faster than BSVM. Runtimes are compared for one of the datasets having annotations from Gene Ontology's complete *biological process *hierarchy (in particular, we used *D*_16_, which is annotated with 629 classes). Run on a cluster of AMD Opteron processors (1.8 - 2.4 GHz, ≥ 2 GB RAM), CLUS-HMC-ENS required 15.9 hours, while SVM-light [44], which is the first step of BSVM, required 190.5 hours for learning the models (i.e., CLUS-HMC-ENS is faster by a factor 12 in this case).

#### Comparison between CLUS-HMC-ENS and the methods in the MouseFunc challenge

In this section we compare CLUS-HMC-ENS to the seven systems that submitted predictions to the MouseFunc challenge. These systems are the ensemble extension of BSVM [18] (which we will call BSVM^+^), Kernel Logistic Regression [13] (which we will call KLR), calibrated SVMs [19] (which we will call CSVM), GENEFAS [4], GENEMANIA [15], the combined functional network and classifier strategy of Kim et al. [16] (which we will call KIM) and the Funckenstein system [17]. These methods were described in the "Related work" section. Note that, when comparing the results, one should keep in mind that each team independently constructed a dataset, possibly using different features. As a result, the differences in performance can be due not only to the learning methods compared, but also the different feature sets used by the methods. As mentioned in the "Datasets" section, the representation that we use is the one of the BSVM^+ ^team.

The organizers have made available a program that computes several evaluation measures and was used to compare the results by the different participating teams in the challenge. This software is available at the same URL where the data can be found, and computes AUROC scores and precision values at several levels of recall for a list of GO terms.

A close inspection of this program reveals that it exhibits some undesirable behaviour. This can easily be verified by observing the result for a classifier that always predicts the same value. The correct function-wise PR curve for any GO term would be a straight line parallel to the recall axis, with precision equal to the frequency of the term. However, the PR curve returned by the software differs from this. If the ordering in which the genes are processed happens to start with a positive gene, then the precision at zero recall equals one. Moreover, if the ordering ends with a negative gene, the precision at recall one is still higher than the class frequency. The ordering in which the examples are processed should be independent from the resulting PR curve.

For this reason, we included the computation of precision and recall in the Clus software. Because the MouseFunc website lists a prediction matrix (containing for each gene-term pair the corresponding probability that the gene is annotated with the GO term) for each of the methods we compare to, we can run our own evaluation program on these predictions, producing corrected results for these methods.

Each method gives predictions for 2815 selected GO terms. These terms are divided into 12 disjunct subsets corresponding to all combinations of the three GO branches (Biological Process, Molecular Function and Cellular Component) with four ranges of specificity, which is defined as the number of genes in the training set to which each term is annotated (3-10, 11-30, 31-100 and 101-300). We have adopted the same subsets and trained and evaluated our models on each of them. Since 1846 of the selected 2815 GO terms were used as annotation in the test set, our evaluation of all the systems is based only on those.

Table 1 shows the AU() results of all the methods on the 12 subsets. Looking at the wins/losses for each of the 12 subsets, according to the (two-sided) Wilcoxon signed rank test, the performance of CLUS-HMC-ENS is significantly better at the 1% level than BSVM^+^(*p *= 4.88·10^-4^), CSVM (*p *= 1.47·10^-3^), GENEFAS (*p *= 4.88·10^-4^), and KIM (*p *= 4.88·10^-4^). CLUS-HMC-ENS has more wins than KLR (*p *= 1.61·10^-2^) and GENEMANIA (*p *= 1.61·10^-2^), but is not significantly better at 1%. CLUS-HMC-ENS is performing significantly worse than Funckenstein (*p *= 9.28·10^-3^).

**Table 1:** Comparison of AU() between Clus-HMC-Ens and the MouseFunc systems

Subset	CLUS-HMC-ENS	**BSVM**^+^	KLR	CSVM	GENEFAS	GeneMANIA	KIM	Funckenstein
BP_3-10	0.045	0.040⊖	0.028⊖	0.029⊖	0.028⊖	0.071⊕	0.029⊖	0.085⊕
BP_11-30	0.055	0.042⊖	0.053	0.017⊖	0.012⊖	0.038⊖	0.031⊖	0.083⊕
BP_31-100	0.109	0.100⊖	0.135⊕	0.077⊖	0.033⊖	0.035⊖	0.044⊖	0.190⊕
BP_101-300	0.173	0.161⊖	0.174⊕	0.146⊖	0.078⊖	0.055⊖	0.051⊖	0.225⊕

CC_3-10	0.182	0.076⊖	0.060⊖	0.046⊖	0.050⊖	0.131⊖	0.128⊖	0.202⊕
CC_11-30	0.207	0.085⊖	0.128⊖	0.094⊖	0.038⊖	0.068⊖	0.112⊖	0.167⊖
CC_31-100	0.233	0.163⊖	0.161⊖	0.074⊖	0.107⊖	0.046⊖	0.127⊖	0.226⊖
CC_101-300	0.220	0.166⊖	0.225⊕	0.157⊖	0.110⊖	0.101⊖	0.094⊖	0.248⊕

MF_3-10	0.266	0.243⊖	0.191⊖	0.205⊖	0.174⊖	0.359⊕	0.189⊖	0.368⊕
MF_11-30	0.356	0.258⊖	0.285⊖	0.275⊖	0.136⊖	0.270⊖	0.215⊖	0.384⊕
MF_31-100	0.360	0.245⊖	0.294⊖	0.231⊖	0.120⊖	0.284⊖	0.191⊖	0.482⊕
MF_101-300	0.368	0.283⊖	0.331⊖	0.386⊕	0.184⊖	0.202⊖	0.140⊖	0.485⊕

For each of the 12 subsets, the AU() of CLUS-HMC-ENS is compared with the MouseFunc systems. A win (⊕) means that the MouseFunc system outperforms CLUS-HMC-ENS, a loss (⊖) means that it is outperformed by CLUS-HMC-ENS.

Table 2 shows the same comparison, but now for . According to the Wilcoxon signed rank test, CLUS-HMC-ENS is performing significantly better at the 1% level than KIM (*p *= 4.88·10^-4^), while it is not significantly different from BSVM^+ ^(*p *= 4.70·10^-1^), KLR (*p *= 1.61·10^-2^), CSVM (*p *= 1.51·10^-1^) and GENEFAS (*p *= 2.59·10^-2^). CLUS-HMC-ENS is performing significantly worse than GENEMANIA (*p *= 9.28·10^-3^) and Funckenstein (*p *= 9.77·10^-4^).

**Table 2:** Comparison of  between CLUS-HMC-ENS and the MouseFunc systems

Subset	CLUS-HMC-ENS	**BSVM**^+^	KLR	CSVM	GENEFAS	GENEMANIA	KIM	Funckenstein
BP_3-10	0.120	0.156⊕	0.075⊖	0.075⊖	0.108⊖	0.170⊕	0.108⊖	0.198⊕
BP_11-30	0.110	0.141⊕	0.087⊖	0.085⊖	0.074⊖	0.151⊕	0.107⊖	0.162⊕
BP_31-100	0.139	0.172⊕	0.158⊕	0.140⊕	0.094⊖	0.177⊕	0.116⊖	0.244⊕
BP_101-300	0.171	0.172⊕	0.169⊖	0.173⊕	0.104⊖	0.160⊖	0.056⊖	0.214⊕

CC_3-10	0.319	0.249⊖	0.119⊖	0.083⊖	0.233⊖	0.324⊕	0.271⊖	0.316⊖
CC_11-30	0.260	0.194⊖	0.212⊖	0.151⊖	0.131⊖	0.235⊖	0.178⊖	0.267⊕
CC_31-100	0.217	0.232⊕	0.197⊖	0.161⊖	0.191⊖	0.261⊕	0.144⊖	0.287⊕
CC_101-300	0.244	0.217⊖	0.259⊕	0.221⊖	0.177⊖	0.258⊕	0.118⊖	0.279⊕

MF_3-10	0.320	0.441⊕	0.258⊖	0.228⊖	0.427⊕	0.465⊕	0.304⊖	0.472⊕
MF_11-30	0.356	0.373⊕	0.347⊖	0.393⊕	0.350⊖	0.401⊕	0.302⊖	0.455⊕
MF_31-100	0.269	0.289⊕	0.230⊖	0.278⊕	0.242⊖	0.291⊕	0.255⊖	0.416⊕
MF_101-300	0.322	0.317⊖	0.321⊖	0.374⊕	0.295⊖	0.391⊕	0.172⊖	0.441⊕

For each of the 12 subsets, the  of CLUS-HMC-ENS is compared with the MouseFunc systems. A win (⊕) means that the MouseFunc system outperforms CLUS-HMC-ENS, a loss (⊖) means that it is outperformed by CLUS-HMC-ENS.

Because , the average over all areas under the function-wise ROC curves, was used as evaluation measure in the MouseFunc challenge [22], we report it in Table 3. According to the Wilcoxon signed rank test, CLUS-HMC-ENS is not performing significantly different at the 1% level than KLR (*p *= 9.10·10^-1^), CSVM (*p *= 2.20·10^-2^), GENEFAS (*p *= 5.69·10^-1^) and KIM (*p *= 3.22·10^-2^). CLUS-HMC-ENS is performing significantly worse than BSVM^+ ^(*p *= 4.88·10^-4^), GENEMANIA (*p *= 9.77·10^-4^) and Funckenstein (*p *= 9.77 10^-4^).

**Table 3:** Comparison of  between Clus-HMC-Ens and the MouseFunc systems

Subset	CLUS-HMC-ENS	**BSVM**^+^	KLR	CSVM	GENEFAS	GENEMANIA	KIM	Funckenstein
BP_3-10	0.695	0.808⊕	0.581⊖	0.588⊖	0.715⊕	0.873⊕	0.813⊕	0.790⊕
BP_11-30	0.748	0.808⊕	0.741⊖	0.659⊖	0.767⊕	0.849⊕	0.822⊕	0.796⊕
BP_31-100	0.831	0.874⊕	0.846⊕	0.778⊖	0.780⊖	0.872⊕	0.851⊕	0.880⊕
BP_101-300	0.823	0.853⊕	0.845⊕	0.813⊖	0.733⊖	0.840⊕	0.795⊖	0.838⊕

CC_3-10	0.748	0.845⊕	0.571⊖	0.618⊖	0.782⊕	0.899⊕	0.865⊕	0.837⊕
CC_11-30	0.791	0.873⊕	0.790⊖	0.785⊖	0.834⊕	0.907⊕	0.846⊕	0.850⊕
CC_31-100	0.863	0.896⊕	0.850⊖	0.851⊖	0.783⊖	0.887⊕	0.863	0.849⊖
CC_101-300	0.845	0.873⊕	0.851⊕	0.821⊖	0.750⊖	0.842⊖	0.808⊖	0.867⊕

MF_3-10	0.818	0.887⊕	0.630⊖	0.681⊖	0.850⊕	0.951⊕	0.880⊕	0.879⊕
MF_11-30	0.842	0.903⊕	0.861⊕	0.836⊖	0.865⊕	0.936⊕	0.884⊕	0.909⊕
MF_31-100	0.838	0.888⊕	0.892⊕	0.881⊕	0.843⊕	0.887⊕	0.884⊕	0.903⊕
MF_101-300	0.874	0.904⊕	0.894⊕	0.884⊕	0.843⊖	0.909⊕	0.844⊖	0.918⊕

For each of the 12 subsets, the  of CLUS-HMC-ENS is compared with the MouseFunc systems. A win (⊕) means that the MouseFunc system outperforms CLUS-HMC-ENS, a loss (⊖) means that it is outperformed by CLUS-HMC-ENS.

The fact that CLUS-HMC-ENS performs better according to AU() than to  and  can be explained as follows. The variance function used to select the best tests gives a higher weight to functions at higher levels of the hierarchy (see "Methods" section), causing CLUS-HMC-ENS to perform well especially on those functions. In contrast to  and , which consider each function as equal, the AU() evaluation measure shares the idea of giving a higher penalty to mistakes made for functions at higher levels of the hierarchy.

We can conclude that, in general, the performance of CLUS-HMC-ENS is not significantly different from that of BSVM^+^, which has been evaluated on the same dataset. Moreover, also compared to the other systems, which have used other preprocessing methods, CLUS-HMC-ENS is competitive: only the Funckenstein method and GENEMANIA produce significantly better results on 3 and 2 evaluation measures, respectively. In a function-wise comparison over all 12 subsets (1846 functions in total), CLUS-HMC-ENS still performed better than Funckenstein on 607 (according to AUPRC) and 625 (according to AUROC) functions, while it had an equal score for 98 (AUPRC) and 97 (AUROC) functions. Similarly, it performed better than GENEMANIA on 645/563 functions and had an equal score for 84/88 functions, respectively. This shows that none of the methods is guaranteed to be the best choice for any given function.

This comparison to the methods in the MouseFunc competition suggests that incorporating functional linkage information in the predictions made by an ensemble method can substantially improve its performance. How this could be achieved for CLUS-HMC-ENS will be investigated in further work.

## Conclusions

In this article, we have presented the use of a decision tree learner, called CLUS-HMC, in functional genomics. The learner produces a single tree that predicts, for a given gene, its biological functions from a function classification scheme, such as the Gene Ontology. The main contributions of this work are the introduction of the tree-based ensemble learner CLUS-HMC-ENS and empirical evidence showing that this learner outperforms several state-of-the-art methods on *S. cerevisiae*, *A. thaliana *and *M. musculus *datasets.

First, we have shown that CLUS-HMC outperforms an existing decision tree learner (C4.5H/M) w.r.t. predictive performance. Second, we have shown that the predictive performance boost in regular classification tasks obtained by using ensembles, carries over to the hierarchical multi-label classification context, in which the gene function prediction task is set. Third, by constructing an ensemble of CLUS-HMC-trees, our method outperforms a statistical learner based on SVMs for *S. cerevisiae*, both in predictive performance and in efficiency. Fourth, this ensemble learner is competitive to statistical and network based methods for *M. musculus *data.

To summarize, CLUS-HMC can give additional biological insight in the predictions. Moreover, CLUS-HMC-ENS yields state-of-the-art quality for gene function prediction. The software implementing these methods is easy to use and available online as open-source software. As such, CLUS-HMC(-ENS) is competitive to the current state-of-the-art systems and therefore, we believe it should be considered for making automated predictions in functional genomics.

## Authors' contributions

LS and CV performed the experimental analysis and drafted the manuscript. JS provided expertise about CLUS-HMC and helped revising the manuscript. HB supervised the study and helped drafting the manuscript. DK developed CLUS-HMC-ENS under the supervision of SD. SD also helped in acquiring the datasets used in the study and provided input to various parts of the manuscript. All authors read and approved the final manuscript.
